# Differences in children and adolescents with SARS-CoV-2 infection: a cohort study in a Brazilian tertiary referral hospital

**DOI:** 10.6061/clinics/2021/e3488

**Published:** 2021-11-17

**Authors:** Heloisa Helena de Sousa Marques, Maria Fernanda Badue Pereira, Angélica Carreira dos Santos, Thais Toledo Fink, Camila Sanson Yoshino de Paula, Nadia Litvinov, Claudio Schvartsman, Artur Figueiredo Delgado, Maria Augusta Bento Cicaroni Gibelli, Werther Brunow de Carvalho, Vicente Odone, Uenis Tannuri, Magda Carneiro-Sampaio, Sandra Grisi, Alberto José da Silva Duarte, Leila Antonangelo, Rossana Pucineli Vieira Francisco, Thelma Suely Okay, Linamara Rizzo Batisttella, Carlos Roberto Ribeiro de Carvalho, Alexandra Valéria Maria Brentani, Clovis Artur Silva, HC-FMUSP Pediatric COVID Study Group, Adriana Pasmanik Eisencraft, Alfio Rossi, Alice Lima Fante, Aline Pivetta Cora, Amelia Gorete A. de Costa Reis, Ana Paula Scoleze Ferrer, Anarella Penha Meirelles de Andrade, Andreia Watanabe, Angelina Maria Freire Gonçalves, Aurora Rosaria Pagliara Waetge, Camila Altenfelder Silva, Carina Ceneviva, Carolina dos Santos Lazari, Deipara Monteiro Abellan, Emilly Henrique dos Santos, Ester Cerdeira Sabino, Fabíola Roberta Marim Bianchini, Flávio Ferraz de Paes Alcantara, Gabriel Frizzo Ramos, Gabriela Nunes Leal, Isadora Souza Rodriguez, João Renato Rebello Pinho, Jorge David Avaizoglou Carneiro, Jose Albino Paz, Juliana Carvalho Ferreira, Juliana Ferreira Ferranti, Juliana de Oliveira Achili Ferreira, Juliana Valéria de Souza Framil, Katia Regina da Silva, Kelly Aparecida Kanunfre, Karina Lucio de Medeiros Bastos, Karine Vusberg Galleti, Lilian Maria Cristofani, Lisa Suzuki, Lucia Maria Arruda Campos, Maria Beatriz de Moliterno Perondi, Maria de Fatima Rodrigues Diniz, Maria Fernanda Mota Fonseca, Mariana Nutti de Almeida Cordon, Mariana Pissolato, Marina Silva Peres, Marlene Pereira Garanito, Marta Imamura, Mayra de Barros Dorna, Michele Luglio, Mussya Cisotto Rocha, Nadia Emi Aikawa, Natalia Viu Degaspare, Neusa Keico Sakita, Nicole Lee Udsen, Paula Gobi Scudeller, Paula Vieira de Vincenzi Gaiolla, Rafael da Silva Giannasi Severini, Regina Maria Rodrigues, Ricardo Katsuya Toma, Ricardo Iunis Citrangulo de Paula, Patricia Palmeira, Silvana Forsait, Sylvia Costa Lima Farhat, Tânia Miyuki Shimoda Sakano, Vera Hermina Kalika Koch, Vilson Cobello

**Affiliations:** Hospital das Clinicas HCFMUSP, Faculdade de Medicina, Universidade de Sao Paulo, Sao Paulo, SP, BR.; Hospital das Clinicas HCFMUSP, Faculdade de Medicina, Universidade de Sao Paulo, SP, BR.

**Keywords:** COVID-19, Children, Adolescent, Outcome, Chronic Disease, Multisystem Inflammatory Syndrome

## Abstract

**OBJECTIVES:**

To compare demographic/clinical/laboratory/treatments and outcomes among children and adolescents with laboratory-confirmed coronavirus disease 2019 (COVID-19).

**METHODS:**

This was a cross-sectional study that included patients diagnosed with pediatric COVID-19 (aged <18 years) between April 11, 2020 and April 22, 2021. During this period, 102/5,951 (1.7%) of all admissions occurred in neonates, children, and adolescents. Furthermore, 3,962 severe acute respiratory syndrome coronavirus 2 (SARS-CoV-2) detection samples were processed in patients aged <18 years, and laboratory-confirmed COVID-19 occurred in 155 (4%) inpatients and outpatients. Six/155 pediatric patients were excluded from the study. Therefore, the final group included 149 children and adolescents (n=97 inpatients and 52 outpatients) with positive SARS-CoV-2 results.

**RESULTS:**

The frequencies of sore throat, anosmia, dysgeusia, headache, myalgia, nausea, lymphopenia, pre-existing chronic conditions, immunosuppressive conditions, and autoimmune diseases were significantly reduced in children and adolescents (*p*<0.05). Likewise, the frequencies of enoxaparin use (*p*=0.037), current immunosuppressant use (*p*=0.008), vasoactive agents (*p*=0.045), arterial hypotension (*p*<0.001), and shock (*p*=0.024) were significantly lower in children than in adolescents. Logistic regression analysis showed that adolescents with laboratory-confirmed COVID-19 had increased odds ratios (ORs) for sore throat (OR 13.054; 95% confidence interval [CI] 2.750-61.977; *p*=0.001), nausea (OR 8.875; 95% CI 1.660-47.446; *p*=0.011), and lymphopenia (OR 3.575; 95% CI 1.355-9.430; *p*=0.010), but also had less hospitalizations (OR 0.355; 95% CI 0.138-0.916; *p*=0.032). The additional logistic regression analysis on patients with preexisting chronic conditions (n=108) showed that death as an outcome was significantly associated with pediatric severe acute respiratory syndrome (SARS) (OR 22.300; 95% CI 2.341-212.421; *p*=0.007) and multisystem inflammatory syndrome in children (MIS-C) (OR 11.261; 95% CI 1.189-106. 581; *p*=0.035).

**CONCLUSIONS:**

Half of the laboratory-confirmed COVID-19 cases occurred in adolescents. Individuals belonging to this age group had an acute systemic involvement of SARS-CoV-2 infection. Pediatric SARS and MIS-C were the most important factors associated with the mortality rate in pediatric chronic conditions with COVID-19.

## INTRODUCTION

A pandemic caused by the novel severe acute respiratory syndrome coronavirus 2 (SARS-CoV-2) was declared by the World Health Organization in March 2020. Children and adolescents with this infection usually present with milder infections and a better prognosis than adults (1-3).

Coronavirus disease 2019 (COVID-19) in pediatric populations accounts for up to 14% of all laboratory-confirmed cases (4). The spectrum of COVID-19 in children and adolescents ranges from asymptomatic to acute critical conditions. Death has rarely been reported and is mainly associated with pediatric severe acute respiratory syndrome (SARS) and multisystem inflammatory syndrome in children (MIS-C) (1-3,5-9).

Preexisting chronic conditions and comorbidities appear to be a risk factor for severe SARS-CoV-2 infection, with high death rates among children and adolescents (5,10). A case series involving pediatric populations of inpatients and outpatients in tertiary/quaternary hospitals for COVID-19 was reported in New York (11-13), Mexico (14), Brazil (2,3,7), Italy (15), Poland (16), the United Kingdom (17), Indonesia (18), and China (19), with a focus on risk factors for severity (2,11,12) and immunosuppression (7,14).

In addition, transmission of SARS-CoV-2 infection occurs more easily in secondary/high school adolescents than in primary school children (20). A recent study demonstrated increased hospitalization rates among adolescents, and approximately one-third of cases were admitted to pediatric intensive care units (PICUs), and 5% required invasive mechanical ventilation (21). However, to the best of our knowledge, few studies have evaluated the differences between children and adolescents with laboratory-confirmed SARS-CoV-2 infection, particularly in inpatients and outpatients followed up in a tertiary referral hospital.

Therefore, the objective of this study was to compare demographic, anthropometric, and clinical data, pediatric preexisting chronic conditions and comorbidities, laboratory tests, imaging abnormalities, treatments, and outcomes among children and adolescents with laboratory-confirmed COVID-19.

## METHODS

Ethics Committee name and study protocol number: HCFMUSP - CAEE 4.889.659.

This was a cross-sectional study that included pediatric patients diagnosed with laboratory-confirmed COVID-19 between April 11, 2020, and April 22, 2021. The study site was the Hospital das Clínicas da Faculdade de Medicina da Universidade de São Paulo (HCFMUSP), Brazil. This university referral hospital is the largest public and teaching hospital complex in Latin America (22-24) and worked as the main reference for pediatric patients with COVID-19 during the pandemic. It attended to patients from four institutes: Instituto Central, Instituto da Criança e do Adolescente, Instituto de Tratamento do Câncer Infantil, and Instituto do Coração, HCFMUSP.

The inclusion criteria were: 1) symptomatic inpatients and outpatients, 2) presence of clinical criteria for the screening of SARS-CoV-2 infection, and 3) age ≤18 years. The exclusion criteria included pregnant adolescents and subjects with incomplete medical record data. Children were defined as those aged <10 years, and adolescents were defined as those aged between ≥10 and <18 years.

The clinical criteria for screening of SARS-CoV-2 infection in children and adolescents were the presence of at least one of the following signs and symptoms during the COVID-19 pandemic: a) influenza-like disease in high-risk children (aged <5 years and/or with underlying conditions); b) pediatric SARS; c) fever without a source; d) fever and rash; e) fever in immunocompromised patients and without any other cause; f) MIS-C; and g) gastrointestinal or neurological signs or symptoms.

Between April 11, 2020 and April 22, 2021, 5,951 pediatric and adult patients with laboratory-confirmed COVID-19were hospitalized. Of these, 102/5,951 (1.7%) of the admissions occurred in neonates, children, and adolescents.

During this period, 3,962 SARS-CoV-2 detection samples were also processed in patients aged <18 years, and laboratory-confirmed COVID-19 occurred in 155 (4%) inpatients and outpatients. Six of the 155 pediatric patients with laboratory-confirmed COVID-19 were excluded because of incomplete medical records (n=3), pregnancy (n=2), and asymptomatic results confirmed by the pre-surgical evaluation (n=1).

Therefore, the final study group comprised 149 children and adolescents (n=97 inpatients and n=52 outpatients) with positive SARS-CoV-2 results: 122/149 (82%) were confirmed using real-time reverse transcription polymerase chain reaction (RT-PCR) assay on nasopharynx and oropharynx secretions, 23/149 (15%) had positive serological tests for SARS-CoV-2, and 4/149 (3%) children and adolescents were positive in both tests. Of these, 119/149 (80%) pediatric patients were diagnosed with COVID-19 in 2020, and 30/149 (20%) were diagnosed between January and April 22, 2021.

This study was approved by the Ethical Committee of our university hospital.

### Data collection and study definitions

Data were systematically retrieved from the medical records of each pediatric patient. The list of children and adolescents was revised by the Research Electronic Data Capture database of information for all pediatric cases with suspected COVID-19 at HCFMUSP, as well as the number of pediatric and adult outpatients with COVID-19 during the study period.

#### 1) Demographics, anthropometric and clinical data

The demographic data of patients with laboratory-confirmed COVID-19 included their current age, sex, and race. Body mass index values were calculated according using the patient’s body weight divided by the square of their height and expressed in units of kg/m^2^. The following clinical manifestations in the pediatric diagnosis of COVID-19 were assessed: duration of signs or symptoms before diagnosis, fever, fever without a source, nasal discharge, sneezing, cough, sore throat, anosmia, dysgeusia, headache, myalgia, arthralgia, conjunctivitis, dyspnea, hypoxemia, nausea, vomiting, diarrhea, cutaneous rash, neurological abnormalities, and pneumonia. Pediatric SARS was defined according to the presence of a flu-like syndrome and at least one of the following: dyspnea, oxygen saturation below 95% in room air, or signs of respiratory distress (2). MIS-C diagnosis was established according to the Centers for Disease Control criteria (25). The severity of pediatric COVID-19 was classified as mild, moderate, severe, or critical according to Dong et al. (26).

#### 2) Preexisting pediatric chronic conditions and comorbidities

Preexisting pediatric chronic conditions in patients with laboratory-confirmed COVID-19 were defined according to whether the signs or symptoms lasted for more than three months, the diagnosis established by the physicians’ scientific knowledge, and according to valid methods or instruments based on specific pediatric diagnostic criteria (27-29).

#### 3) Laboratory tests, imaging abnormalities, treatments and outcomes

Laboratory tests at the time of COVID-19 diagnosis included assessments of: hemoglobin concentration; leukocyte, neutrophil, lymphocyte, and thrombocyte counts; inflammatory biomarkers (C-reactive protein, erythrocyte sedimentation rate, fibrinogen, D- dimer, and ferritin); lactate dehydrogenase; serum albumin; aspartate and alanine aminotransferase; gamma-glutamyl transferase; alkaline phosphatase; blood urea; serum creatinine; triglycerides; muscle and brain creatine kinase and creatine kinase-isoenzymes; troponin T; prothrombin times; international normalized ratios; activated partial thromboplastin times; and hematuria, proteinuria, and pyuria. The examinations were conducted at the Clinical Laboratory of the Instituto Central, HCFMUSP.

Chest radiography and computed tomography were performed by a pediatric radiologist at the Instituto da Criança e do Adolescente do HCFMUSP, to search for abnormalities suggestive of SARS-CoV-2-related lesions, such as ground-glass opacities, consolidation, and linear and reticular opacities. Echocardiographic abnormalities were detected by two pediatric specialists at the Instituto da Criança e do Adolescente do HCFMUSP; abnormalities suggestive of SARS-CoV-2-related lesions, such as myocardial dysfunction, myocarditis, pericarditis, and/or coronary artery aneurysm z-scores ≥2.5, were identified.

Data on factors, such as transfusion of blood components (red blood, platelets, and plasma), oxygen therapy, concomitant antibiotics, oseltamivir, intravenous immunoglobulin, enoxaparin, aspirin, glucocorticoid use, intravenous methylprednisolone pulse therapy, dialysis, current use of immunosuppressants, and chemotherapy for cancer, were systematically recorded.

The following outcomes were also systematically evaluated in children and adolescents who were admitted to the hospital with COVID-19: hospitalization, duration of ward hospitalization, duration of PICU hospitalization, mechanical ventilation, duration of mechanical ventilation, use of vasoactive agents, arterial hypotension, shock, disseminated intravascular coagulation, thrombosis, and death.

#### 4) Molecular and serological tests for SARS-CoV-2 detection

SARS-CoV-2 infection was confirmed by real-time RT-PCR or antibody testing. Real-time RT-PCR to assess SARS-CoV-2 genes was performed on respiratory samples at the molecular biology laboratory of our hospital (30). Tests for antibodies against the S proteins from the coronavirus spike were conducted in the Laboratório de Imunologia do Instituto Central do HCFMUSP using the following two assays: an enzyme-linked immunosorbent assay for detecting anti-SARS-CoV-2 immunoglobulin G (IgG) antibodies and a rapid immunochromatographic assay for detecting anti-SARS-CoV-2 IgM and IgG antibodies (31,32).

### Statistical analyses

Bivariate analyses were performed to compare preexisting pediatric chronic conditions among children and adolescents with laboratory-confirmed COVID-19. A non-parametric test (Mann-Whitney *U* test) or a parametric test (Student’s *t*-test) was used for continuous variables, and the results are presented as medians (minimum and maximum values) or means±standard deviations, respectively. Fisher’s exact test or the Chi-square test was used for categorical variables. Logistic regression analyses were performed with age (<10 years and ≥10 years) or death as the dependent variable and independent variables that presented a statistical significance of *p*<0.2 in the bivariate analyses. The level of significance was established at 5%.

## RESULTS

Table 1 includes the demographic, anthropometric, and clinical data, as well as the preexisting pediatric chronic conditions and comorbidities present in children *vs.* those in adolescents with laboratory-confirmed COVID-19. The median duration of signs and symptoms before diagnosis was significantly lower in children than in adolescents [2 (0-60) *vs.* 3 (0-90) days, *p=*0.027]. The frequencies of sore throat, anosmia, dysgeusia, headache, myalgia, nausea, pediatric preexisting chronic conditions, autoimmune diseases, and the use of immunosuppressants were significantly reduced in children compared with adolescents (*p<*0.05, Table 1). No differences in the severity of pediatric COVID-19 were observed between the groups (*p=*0.287, Table 1).

Table 2 illustrates the laboratory tests and imaging abnormalities in children and adolescents with laboratory-confirmed COVID-19. The median lymphocyte [2,610 (0-17,860) *vs.* 1,315 (0-9,890)/mm^3^, *p<*0.001] and thrombocyte counts (254,439±136,531 *vs.* 197,054±115,116/mm^3^, *p=*0.003) were significantly higher in children than in adolescents, whereas fibrinogen [284 (0-766) *vs.* 388.5 (0-1,274) mg/dL, *p=*0.023] and ferritin levels [118 (0-15,179) *vs.* 358 (37-35,976) ng/mL, *p=*0.016] were significantly reduced in the first group (Table 2).

A comparison of the treatments and outcomes between children and adolescents with laboratory-confirmed COVID-19 are shown in Table 3. The frequencies of enoxaparin (8% *vs.* 20%, *p=*0.037), current immunosuppressants (19% *vs.* 39%, *p=*0.008), vasoactive agents (4% *vs.* 13%, *p=*0.045), arterial hypotension (0% *vs.* 16%, *p<*0.001), and shock (7% *vs.* 15%, *p=*0.024) were significantly lower in children than in adolescents, whereas hospitalization was significantly higher in children (74% *vs.* 56%, *p=*0.016).

**Figure 1:**
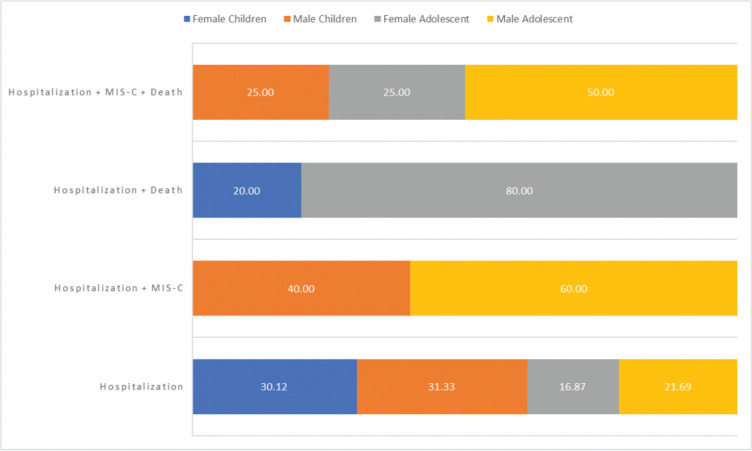
Demographic data and outcomes in children (aged <10 years) *vs.* those in adolescents (aged ≥10 years) with laboratory-confirmed coronavirus disease 2019 (COVID-19). MIS-C, multisystem inflammatory syndrome in children; hospitalization+MIS-C+death (*p=*0.787), hospitalization+death (*p=*0.019), hospitalization+MIS-C (*p=*0.133), and hospitalization (*p=*0.004).

Figure 1 summarizes the demographic data and outcomes of children *vs.* those in adolescents with laboratory-confirmed COVID-19. The frequency of hospitalization and death was significantly lower in female children than in female adolescents (20% *vs.* 80%, *p=*0.019). The frequency of hospitalization was significantly higher in female and male children than in female and male adolescents (30% *vs.* 31% *vs.* 17% *vs.* 22%, *p=*0.004). No differences in other demographic data or in the combination of outcomes were observed (*p*>0.05, Figure 1).

Logistic regression analysis showed that adolescents with laboratory-confirmed COVID-19 had increased odds ratios (ORs) of sore throat (OR 13.054; 95% confidence interval [CI] 2.750-61.977; *p=*0.001), nausea (OR 8.875; 95% CI 1.660-47.446; *p=*0.011), and lymphopenia (OR 3.575; 95% CI 1.355-9.430; *p=*0.010), although they also had fewer hospital admissions (OR 0.355; 95% CI 0.138-0.916; *p=*0.032).

A further logistic regression analysis, which included death as the dependent variable and age, pediatric SARS, MIS-C, and preexisting chronic conditions as the independent variables, showed that the presence of death was significantly associated with pediatric SARS (OR 34.269; 95% CI 3.754-312.802; *p=*0.002) and MIS-C (OR 11.555; 95% CI 1.478-90.327; *p=*0.020). An additional logistic regression analysis (including only patients with preexisting chronic conditions (n=108), which used death as the dependent variable and age, pediatric SARS, and MIS-C as the independent variables, showed that the presence of death was significantly associated with pediatric SARS (OR 22.300; 95% CI 2.341-212.421; *p=*0.007) and MIS-C (OR 11.261; 95% CI 1.189-106.581; *p=*0.035).

## DISCUSSION

This study showed that laboratory-confirmed COVID-19 occurred in 50% of the studied adolescents. Individuals belonging to this age group had more intense multisystemic involvement of SARS-CoV-2 infection than children, mainly presenting with sore throat, nausea, and lymphopenia. Pediatric SARS and MIS-C were the most important factors associated with mortality in patients with chronic diseases and COVID-19.

The main strength of this study was the inclusion of pediatric patients with laboratory-confirmed SARS-CoV-2 infection, involving almost 80% of pediatric chronic conditions. Another strength was the use of a standardized database to minimize bias. Our tertiary university hospital is the largest public funded in Latin America (2,3,22). During the COVID-19 pandemic in Sao Paulo, which is the most populous city in Brazil, this academic health complex, through its Crisis Management Committee, guided the hospitalization of pediatric and adult patients with moderate to critical COVID-19. This large hospital complex provided exclusive care for inpatients with COVID-19, with a total of 900 beds and more than 300 ICU beds for adults and children/adolescents in the peak months and during the pandemic waves (23,24,33). Another advantage of this study was the pediatric multidisciplinary team that was available to support inpatients and outpatients with COVID-19; it involved physicians and fellows of various pediatric subspecialties, as well as nurses, physiotherapists, psychologists, nutritionists, biologists, social workers, clinical pharmacists, and physical educators. This group was selected to attend and to conduct specific research involving neonates, children, and adolescent inpatients and outpatients with SARS-CoV-2 infection (2,4,6-9,34-38), as well as the implementation of an innovative telemedicine program that was established during the COVID-19 pandemic (39).

We confirmed that hospitalizations because of COVID-19 affected the pediatric population less frequently than adults, as has been previously reported (14). In fact, admission for pediatric COVID-19 occurred in 1.7% of the evaluated patients during one year of the pandemic in our university hospital. Interestingly, only 4% of them had laboratory-confirmed COVID-19 of a total of almost 4,000 samples from patients presenting with warning signs for the screening of SARS-CoV-2 infection, reinforcing the rarity of this infection during this year in our pediatric population.

We extend the previous reports of laboratory-confirmed pediatric COVID-19 cases, where adolescents had a more intense, acute symptomatic, and systemic involvement of COVID-19 than children but also a lower rate of hospitalization. The higher frequencies of sore throat and nausea, as well as anosmia, dysgeusia, headache, and myalgia, in adolescents with SARS-CoV-2 infection may be related to the high reporting rates of these signs and symptoms in the individuals belonging to this age group, as they are more aware of COVID-19 signs and symptoms than younger children. Furthermore, preexisting chronic conditions, particularly the use of immunosuppressants and presence of autoimmune diseases, appear to have contributed to the clinical spectrum of COVID-19 in adolescents (1,2,4,8,33,36).

Furthermore, lymphopenia is a distinct laboratory feature of adolescents with COVID-19. This finding may be related to the higher frequencies of immunosuppressive conditions observed in our teenagers at the time of this emerging infectious disease diagnosis, a possible relationship with the disease activity, or the use of immunosuppressants or chemotherapy drugs. A recent pediatric COVID-19 meta-analysis revealed that lymphopenia and leukopenia were the most common white cell abnormalities (40).

Shock and arterial hypotension are striking complications of the disease course in adolescents with SARS-CoV-2 infection. Individuals belonging to this age group also required more enoxaparin and vasoactive agents, indicating a critical COVID-19 condition among adolescents.

Importantly, our data showed a mortality rate of 6%, similar to that in other studies in tertiary hospitals that ranged from 0% to 5% (12,14,16,18). In contrast, a cross-sectional study conducted in the first semester of 2020 using Brazilian data from the National Epidemiological Surveillance Information System showed a higher death rate of 15% in 2,570 confirmed pediatric COVID-19 cases with SARS (5).

Pediatric SARS and MIS-C were the most important causes of death in both children and adolescents with COVID-19, as has also been reported in other studies (2,18). Reinforcing these results, the autopsy features of five of our patients with SARS-CoV-2 infection showed primary pulmonary disease, with SARS and diffuse alveolar damage present in some patients and MIS-C with multiple organ involvement in another patient (41). Interestingly, the isolation of SARS-CoV-2 with cellular ultrastructural changes in several organs of patients with MIS-C supports the idea of a direct effect of SARS-CoV-2 on tissues and relevant role of this agent in the pathogenesis of this severe COVID-19 involvement (41,42).

Our study has limitations, such as a population selection bias resulting from the study being conducted in a tertiary referral hospital for children, including patients with a more severe spectrum of COVID-19. In addition, there were no data on cytokine levels, which may have played a role in the outcomes of patients with COVID-19 and MIS-C. The SARS-CoV-2 lineage P.1 (gamma variant of concern), which was discovered in Manaus, Brazil in early January 2021, was responsible for the second wave across the country and was not evaluated in this study (43,44). However, only 20% of our cases were diagnosed in the first four months of 2021. In fact, during that period, COVID-19 vaccination was initiated in adults; thus, this may have contributed to the decrease in SARS-CoV-2 transmission in communities, families, and hospital environments.

In conclusion, laboratory-confirmed COVID-19 occurred in 50% of the adolescents in this study. Adolescents have a more intense, acute, and systemic involvement of SARS-CoV-2 infection. Pediatric SARS and MIS-C were the most important factors associated with the mortality rates in pediatric chronic conditions with COVID-19.

### HC-FMUSP Pediatric COVID Study Group

Adriana Pasmanik Eisencraft, Alfio Rossi Junior, Alice Lima Fante, Aline Pivetta Cora, Amelia Gorete A. de Costa Reis, Ana Paula Scoleze Ferrer, Anarella Penha Meirelles de Andrade, Andreia Watanabe, Angelina Maria Freire Gonçalves, Aurora Rosaria Pagliara Waetge, Camila Altenfelder Silva, Carina Ceneviva, Carolina dos Santos Lazari, Deipara Monteiro Abellan, Emilly Henrique dos Santos, Ester Cerdeira Sabino, Fabíola Roberta Marim Bianchini, Flávio Ferraz de Paes Alcantara, Gabriel Frizzo Ramos, Gabriela Nunes Leal, Isadora Souza Rodriguez, João Renato Rebello Pinho, Jorge David Avaizoglou Carneiro, Jose Albino Paz, Juliana Carvalho Ferreira, Juliana Ferreira Ferranti, Juliana de Oliveira Achili Ferreira, Juliana Valéria de Souza Framil, Katia Regina da Silva, Kelly Aparecida Kanunfre, Karina Lucio de Medeiros Bastos, Karine Vusberg Galleti, Lilian Maria Cristofani, Lisa Suzuki, Lucia Maria Arruda Campos, Maria Beatriz de Moliterno Perondi, Maria de Fatima Rodrigues Diniz, Maria Fernanda Mota Fonseca, Mariana Nutti de Almeida Cordon, Mariana Pissolato, Marina Silva Peres, Marlene Pereira Garanito, Marta Imamura, Mayra de Barros Dorna, Michele Luglio, Mussya Cisotto Rocha, Nadia Emi Aikawa, Natalia Viu Degaspare, Neusa Keico Sakita, Nicole Lee Udsen, Paula Gobi Scudeller, Paula Vieira de Vincenzi Gaiolla, Rafael da Silva Giannasi Severini, Regina Maria Rodrigues, Ricardo Katsuya Toma, Ricardo Iunis Citrangulo de Paula, Patricia Palmeira, Silvana Forsait, Sylvia Costa Lima Farhat, Tânia Miyuki Shimoda Sakano, Vera Hermina Kalika Koch, Vilson Cobello Junior. All of the members are from Hospital das Clinicas HCFMUSP, Faculdade de Medicina, Universidade de Sao Paulo, SP, BR.

## AUTHOR CONTRIBUTIONS

All named authors approved the final draft of the manuscript, approved the submission to the journal, and are willing to take responsibility for it in its entirety. All authors contributed substantially to the conception and design of the study and in the analysis and interpretation of the data. All authors revised the manuscript critically and approved its final version.

## Figures and Tables

**Table 1:** Demographic, anthropometric and clinical data and pediatric preexisting chronic conditions in children (aged <10 years) *vs.* those in adolescents (aged ≥10 years) with laboratory-confirmed coronavirus disease 2019 (COVID-19).

Variables	Children (n=74)	Adolescents (n=75)	*p*
Demographic and anthropometric data			
Current age, years	2 (0-9.83)	14.75 (10.1-17.9)	<0.001
Male sex	42 (57)	37 (49)	0.364
Caucasian race	50 (67)	53 (71)	0.715
Body mass index, kg/m^2^ (n=125)	0.18±2.32	0.12±1.76	0.879
Clinical data			
Duration of signs/symptoms before diagnosis, days (n=148)	2 (0-60)	3 (0-90)	0.027
Fever	56 (76)	53 (71)	0.490
Fever without a source	10 (13)	5 (7)	0.165
Duration of fever, days (n=100)	1 (1-8)	2 (1-15)	0.050
Nasal discharge	30 (40)	29 (39)	0.815
Sneezing	14 (19)	11 (15)	0.487
Cough	31 (42)	39 (52)	0.216
Sore throat	2 (3)	22 (29)	<0.001
Anosmia	0 (0)	12 (16)	<0.001
Dysgeusia	0 (0)	6 (8)	0.013
Headache	6 (8)	29 (39)	<0.001
Myalgia	4 (5)	25 (33)	<0.001
Arthralgia	0 (0)	3 (4)	0.082
Conjunctivitis	4 (5)	5 (7)	0.747
Dyspnea	26 (35)	31 (41)	0.436
Hypoxemia	23 (31)	24 (32)	0.904
Nausea	2 (3)	15 (20)	0.001
Vomiting	15 (20)	11 (15)	0.368
Diarrhea	8 (11)	13 (17)	0.253
Cutaneous rash	5 (7)	6 (8)	0.772
Neurological abnormalities	9 (12)	6 (8)	0.399
Pneumonia	16 (22)	23 (31)	0.209
Severe acute respiratory syndrome	12 (16)	17 (23)	0.302
Multisystem inflammatory syndrome in children (MIS-C)	3 (4)	6 (8)	0.312
Pediatric preexisting chronic conditions	53 (72)	65 (87)	0.024
Diabetes mellitus	0 (3)	3 (4)	0.082
Arterial hypertension	6 (8)	9 (12)	0.430
Immunosuppressive conditions	26 (35)	41 (55)	0.017
Inborn errors of immunity	2 (3)	1 (1)	0.552
Solid organ transplantation	1 (1)	5 (7)	0.099
Hematopoietic stem cell transplantation	2 (3)	3 (4)	0.660
Cancer	14 (19)	14 (19)	0.969
Chronic kidney disease	5 (7)	6 (8)	0.772
Autoimmune conditions	3 (4)	10 (13)	0.045
Severity of pediatric COVID-19			
Mild	37 (50)	39 (52)	0.287
Moderate	14 (19)	9 (12)	
Severe	16 (22)	13 (17)	
Critical	7 (9)	14 (19)	

Results are presented as n (%), medians (minimum-maximum values), and means±standard deviations.

**Table 2:** Laboratory tests and imaging abnormalities in children (aged <10 years) *vs.* those in adolescents (≥10 years) with laboratory-confirmed coronavirus disease 2019 (COVID-19).

Variables	Children (n=74)	Adolescents (n=75)	*p*
Hematological parameters			
Hemoglobin concentration, g/dL (n=126)	11.28±2.09	11.55±2.19	0.471
Hemoglobin <10 g/dL (n=126)	17/66 (26)	11/60 (18)	0.317
Leucocyte count/mm^3^ (n=126)	8,210±5,815	7,377±4,583	0.377
Leucopenia <4,000/mm^3^ (n=126)	7/66 (11)	14/60 (23)	0.056
Neutrophil count/ mm^3^ (n=125)	3,200 (0-28,768)	3,905 (0-27,900)	0.064
Neutropenia <1,000/mm^3^ (n=125)	8/65 (12)	5/60 (8)	0.467
Lymphocyte count/mm^3^ (n=125)	2,610 (0-17,860)	1,315 (0-9,890)	<0.001
Lymphopenia <1,500/mm^3^ (n=125)	15/65 (23)	33/60 (55)	<0.001
Thrombocyte count/mm^3^ (n=125)	254,439±136,531	197,054±115,116	0.003
Thrombocytopenia <100,000/ mm^3^ (n=125)	8/66 (12)	13/59 (22)	0.139
Inflammatory markers			
C-reactive protein, mg/L (n=118)	10.96 (0.3-311)	20.8 (0.3-580.4)	0.050
C-reactive protein >30 mg/L (n=118)	21/61 (34)	25/57 (44)	0.294
ESR, mm/first hour (n=23)	36.6 (0-140)	35 (0-140)	0.804
Fibrinogen, mg/dL (n=65)	284 (0-766)	388.5 (0-1,274)	0.023
D-dimer, ng/mL (n=91)	1,286 (190-95,040)	1,043 (190-86,900)	0.584
D-dimer >1000 ng/mL (n=91)	26/45 (58)	24/46 (52)	0.591
Ferritin, ng/mL (n=96)	118 (0-15,179)	358 (37-35,976)	0.016
Other exams			
Lactate dehydrogenase, U/L (n=76)	316.5 (4-3,040)	335.5 (0-6,000)	0.621
Serum albumin, g/dL (n=71)	3.70±0.73	3.42±0.73	0.121
Aspartate aminotransferase, U/L (n=111)	32.5 (13-377)	26 (7-2,002)	<0.001
Alanine aminotransferase, U/L (n=111)	22 (6-495)	24 (5-3,338)	0.885
Gamma-glutamyl transferase, U/L (n=61)	33 (6-1,262)	92.5 (15-483)	0.116
Alkaline phosphatase, U/L (n=58)	163 (77-834)	133 (69-545)	0.038
Blood urea, mg/dL (n=114)	18 (6-106)	23 (4-186)	0.012
Serum creatinine, mg/dL (n=114)	0.34 (0.03-17)	0.56 (0.17-10.06)	<0.001
Triglycerides, mg/dL (n=31)	93 (41-750)	146 (59-308)	0.477
CK, U/L (n=62)	101 (31-1,008)	90 (13-3,331)	0.703
CK-MB, ng/mL (n=52)	2.85 (0.64-14.7)	1.34 (0.3-28.94)	0.010
Troponin T, ng/mL (n=86)	0.011 (0.003-0.08)	0.009 (0.003-1.05)	0.941
Prothrombin time, s (n=92)	13.1 (1.28-16.1)	13.35 (10.8-38.2)	0.061
INR (n=92)	1.01 (0.9-1.23)	1.08 (0.9-3.13)	0.013
Activated partial thromboplastin time, s (n=91)	32.17±7.16	36.28±7.32	0.008
Hematuria >5 erythrocytes/mL (n=66)	6/39 (15)	7/27 (26)	0.290
Proteinuria >0.5 g/day (n=57)	2/34 (6)	4/23 (17)	0.165
Chest X-ray abnormalities (n=90)	25/46 (54)	22/44 (50)	0.680
Pulmonary CT abnormalities (n=41)	15/20 (75)	16/21 (76)	0.929
Cardiac alterations on echocardiogram (n=68)	14/34 (41)	13/34 (38)	0.802

Results are presented as n (%), medians (minimum-maximum values), and means±standard deviations; ESR, erythrocyte sedimentation rate; CK, creatine kinase; CK-MB, muscle and brain creatine kinase-isoenzyme; INR, international normalized ratio; CT, computed tomography.

**Table 3:** Treatments and outcomes in children (aged <10 years) *vs.* those in adolescents (aged ≥10 years) with laboratory-confirmed coronavirus disease 2019 (COVID-19).

Variables	Children (n=74)	Adolescents (n=75)	*p*
Treatments			
Blood product transfusion	9 (12)	11 (15)	0.654
Red blood cell transfusion	9 (12)	10 (13)	0.830
Platelet transfusion	4 (5)	8 (11)	0.238
Plasma transfusion	2 (3)	2 (3)	0.989
Oxygen therapy	23 (31)	27 (36)	0.525
Antibiotics	39 (53)	40 (53)	0.939
Oseltamivir	18 (24)	23 (31)	0.386
Intravenous immunoglobulin	4 (5)	6 (8)	0.527
Enoxaparin	6 (8)	15 (20)	0.037
Aspirin	1 (1)	5 (7)	0.099
Glucocorticoids	15 (20)	17 (23)	0.722
Intravenous methylprednisolone pulse therapy	4 (5)	2 (3)	0.395
Dialysis for acute renal injury or shock	2 (3)	4 (5)	0.414
Current immunosuppressants	14 (19)	29 (39)	0.008
Current chemotherapy for cancer	10 (13)	10 (13)	0.974
Outcomes			
Hospitalization	55 (74)	42 (56)	0.019
Duration of ward hospitalization, days (n=89)	6.5 (1-120)	8 (1-67)	0.813
Duration of PICU hospitalization, (n=30)	5 (1-60)	7 (2-46)	0.620
Mechanical ventilation	7 (9)	13 (17)	0.876
Duration of mechanical ventilation, (n=32)	5 (3-15)	6.5 (2-15)	0.915
Use of vasoactive agents	3 (4)	10 (13)	0.045
Arterial hypotension	0 (0)	12 (16)	<0.001
Shock	5 (7)	11 (15)	0.024
Disseminated intravascular coagulation	0 (0)	5 (7)	0.876
Thrombosis	1 (1)	5 (7)	0.099
Death	2 (3)	7 (9)	0.089

Results are presented in n (%), medians (minimum-maximum values), and means±standard deviations; PICU, pediatric intensive care unit.

## References

[1] Safadi MAP, Silva CAAD (2020). The challenging and unpredictable spectrum of COVID-19 in children and adolescents. Rev Paul Pediatr.

[2] Pereira MFB, Litvinov N, Farhat SCL, Eisencraft AP, Gibelli MABC, Carvalho WB (2020). Severe clinical spectrum with high mortality in pediatric patients with COVID-19 and multisystem inflammatory syndrome. Clinics (Sao Paulo).

[3] Ferreira JC, Ho YL, Besen BAMP, Malbuisson LMS, Taniguchi LU, Mendes PV (2020). Characteristics and outcomes of patients with COVID-19 admitted to the ICU in a university hospital in São Paulo, Brazil - study protocol. Clinics (Sao Paulo).

[4] American Academy of Pediatrics Children and COVID-19: State-Level Data Report.

[5] Sousa BLA, Sampaio-Carneiro M, de Carvalho WB, Silva CA, Ferraro AA (2020). Differences among Severe Cases of Sars-CoV-2, Influenza, and Other Respiratory Viral Infections in Pediatric Patients: Symptoms, Outcomes and Preexisting Comorbidities. Clinics (Sao Paulo).

[6] Diniz MFR, Cardoso MF, Sawamura KSS, Menezes CRB, Lianza AC, Pereira MFB (2021). The Heart of Pediatric Patients with COVID-19: New Insights from a Systematic Echocardiographic Study in a Tertiary Hospital in Brazil. Arq Bras Cardiol.

[B07] de Paula CSY, Palandri GG, Fonseca TS, Vendramini TCA, Farhat SCL, Pereira MFB (2021). Gastrointestinal manifestations are associated with severe pediatric COVID-19: A study in tertiary hospital. J Infect.

[B08] Silva CA, Queiroz LB, Fonseca CB, Silva LEVD, Lourenço B, Marques HHS (2020). Spotlight for healthy adolescents and adolescents with preexisting chronic diseases during the COVID-19 pandemic. Clinics (Sao Paulo).

[9] Tannuri U, Tannuri ACA, Cordon MNA, Miyatani HT (2020). Low incidence of COVID-19 in children and adolescent post-liver transplant at a Latin American reference center. Clinics (Sao Paulo).

[10] Xu PP, Tian RH, Luo S, Zu ZY, Fan B, Wang XM (2020). Risk factors for adverse clinical outcomes with COVID-19 in China: a multicenter, retrospective, observational study. Theranostics.

[11] Zachariah P, Johnson CL, Halabi KC, Ahn D, Sen AI, Fischer A (2020). Epidemiology, Clinical Features, and Disease Severity in Patients With Coronavirus Disease 2019 (COVID-19) in a Children's Hospital in New York City, New York. JAMA Pediatr.

[12] Chao JY, Derespina KR, Herold BC, Goldman DL, Aldrich M, Weingarten J (2020). Clinical Characteristics and Outcomes of Hospitalized and Critically Ill Children and Adolescents with Coronavirus Disease 2019 at a Tertiary Care Medical Center in New York City. J Pediatr.

[13] Biko DM, Ramirez-Suarez KI, Barrera CA, Banerjee A, Matsubara D, Kaplan SL (2021). Imaging of children with COVID-19: experience from a tertiary children's hospital in the United States. Pediatr Radiol.

[14] Macias-Parra M, Fortes-Gutierrez S, Aguilar-Gomez N, Diaz-Garcia L, Otero-Mendoza F, Arias de la Garza E (2021). Clinical and Epidemiological Characteristics of Paediatric Patients Diagnosed with COVID-19 in a Tertiary Hospital in Mexico City. J Trop Pediatr.

[15] Romani L, Chiurchiù S, Santilli V, Bernardi S, Haywood Lombardi M, Scarselli A (2020). COVID-19 in Italian paediatric patients: The experience of a tertiary children's hospital. Acta Paediatr.

[16] Mania A, Mazur-Melewska K, Lubarski K, Kuczma-Napierała J, Mazurek J, Jończyk-Potoczna K (2021). Wide spectrum of clinical picture of COVID-19 in children - From mild to severe disease. J Infect Public Health.

[17] Alders N, Penner J, Grant K, Patterson C, Hassell J, MacDermott N (2020). COVID-19 Pandemic Preparedness in a UK Tertiary and Quaternary Children's Hospital: Tales of the Unexpected. J Pediatric Infect Dis Soc.

[18] Dewi R, Kaswandani N, Karyanti MR, Setyanto DB, Pudjiadi AH, Hendarto A (2021). Mortality in children with positive SARS-CoV-2 polymerase chain reaction test: Lessons learned from a tertiary referral hospital in Indonesia. Int J Infect Dis.

[19] Cai J, Wang X, Zhao J, Ge Y, Xu J, Tian H (2020). Comparison of Clinical and Epidemiological Characteristics of Asymptomatic and Symptomatic SARS-CoV-2 Infection in Children. Virol Sin.

[20] Goldstein E, Lipsitch M, Cevik M (2021). On the Effect of Age on the Transmission of SARS-CoV-2 in Households, Schools, and the Community. J Infect Dis.

[21] Havers FP, Whitaker M, Self JL, Chai SJ, Kirley PD, Alden NB (2021). Hospitalization of Adolescents Aged 12-17 Years with Laboratory-Confirmed COVID-19 - COVID-NET, 14 States, March 1, 2020-April 24, 2021. MMWR Morb Mortal Wkly Rep.

[22] Miethke-Morais A, Perondi B, Harima L, Montal AC, Baldassare RM, Moraes DP (2020). Overcoming barriers to providing comprehensive inpatient care during the COVID-19 pandemic. Clinics (Sao Paulo).

[23] Perondi B, Miethke-Morais A, Montal AC, Harima L, Segurado AC (2020). Setting up hospital care provision to patients with COVID-19: lessons learnt at a 2400-bed academic tertiary center in São Paulo, Brazil. Braz J Infect Dis.

[24] Busatto GF, de Araújo AL, Duarte AJDS, Levin AS, Guedes BF, Kallas EG (2021). Post-acute sequelae of SARS-CoV-2 infection (PASC): a protocol for a multidisciplinary prospective observational evaluation of a cohort of patients surviving hospitalisation in Sao Paulo, Brazil. BMJ Open.

[25] Center for disease and control and prevention (CDC) Information for Healthcare Providers about Multisystem Inflammatory Syndrome in Children (MIS-C).

[26] Dong Y, Mo X, Hu Y, Qi X, Jiang F, Jiang Z (2020). Epidemiological Characteristics of 2143 Pediatric Patients With 2019 Coronavirus Disease in China. Pediatrics.

[27] Alveno RA, Miranda CV, Passone CG, Waetge AR, Hojo ES, Farhat SCL (2018). Pediatric chronic patients at outpatient clinics: a study in a Latin American University Hospital. J Pediatr (Rio J).

[28] Ramos GF, Ribeiro VP, Mercadante MP, Ribeiro MP, Delgado AF, Farhat SCL (2019). Mortality in adolescents and young adults with chronic diseases during 16 years: a study in a Latin American tertiary hospital. J Pediatr (Rio J).

[29] Passone CGB, Grisi SJ, Farhat SC, Manna TD, Pastorino AC, Alveno RA (2019). Complexity of pediatric chronic disease: cross-sectional study with 16,237 patients followed by multiple medical specialties. Rev Paul Pediatr.

[30] Corman VM, Landt O, Kaiser M, Molenkamp R, Meijer A, Chu DK (2020). Detection of 2019 novel coronavirus (2019-nCoV) by real-time RT-PCR. Euro Surveill.

[31] Shen B, Zheng Y, Zhang X, Zhang W, Wang D, Jin J (2020). Clinical evaluation of a rapid colloidal gold immunochromatography assay for SARS-Cov-2 IgM/IgG. Am J Transl Res.

[32] Beavis KG, Matushek SM, Abeleda APF, Bethel C, Hunt C, Gillen S (2020). Evaluation of the EUROIMMUN Anti-SARS-CoV-2 ELISA Assay for detection of IgA and IgG antibodies. J Clin Virol.

[33] Lazar Neto F, Salzstein GA, Cortez AL, Bastos TL, Baptista FVD, Moreira JA (2021). Comparative assessment of mortality risk factors between admission and follow-up models among patients hospitalized with COVID-19. Int J Infect Dis.

[34] Palmeira P, Barbuto JAM, Silva CAA, Carneiro-Sampaio M (2020). Why is SARS-CoV-2 infection milder among children?. Clinics (Sao Paulo).

[B35] Lavorato SSM, Helito AC, Barros VPMFR, Roz DFP, Saccani LP, Martiniano LVM (2021). Assistance and health care provided to adolescents with chronic and immunosuppressive conditions in a tertiary university hospital during the COVID-19 pandemic. Clinics (Sao Paulo).

[B36] Pedrosa T, Kupa LVK, Aikawa NE, Pasoto SG, Bonfá E, Silva CA (2020). Lupus nephritis-related issues during COVID-19 pandemic quarantine. Lupus.

[B37] Ihara BP, Strabelli CA, Simon JR, Viana VS, Sallum AM, Kozu KT (2021). Laboratory-confirmed pediatric COVID-19 in patients with rheumatic diseases: A case series in a tertiary hospital. Lupus.

[38] Carvalho WB, Gibelli MAC, Krebs VLJ, Calil VMLT, Nicolau CM, Johnston C (2020). Neonatal SARS-CoV-2 infection. Clinics (Sao Paulo).

[39] Severini RDSG, Oliveira PC, Couto TB, Simon Junior H, Andrade APM, Nanbu DY (2021). Fast, cheap and feasible: Implementation of pediatric telemedicine in a public hospital during the Covid-19 pandemic. J Pediatr (Rio J).

[40] Toba N, Gupta S, Ali AY, ElSaban M, Khamis AH, Ho SB (2021). COVID-19 under 19: A meta-analysis. Pediatr Pulmonol.

[41] Duarte-Neto AN, Caldini EG, Gomes-Gouvêa MS, Kanamura CT, de Almeida Monteiro RA, Ferranti JF (2021). An autopsy study of the spectrum of severe COVID-19 in children: From SARS to different phenotypes of MIS-C. EClinicalMedicine.

[42] Dolhnikoff M, Ferreira Ferranti J, de Almeida Monteiro RA, Duarte-Neto AN, Soares Gomes-Gouvêa M, Viu Degaspare N (2020). SARS-CoV-2 in cardiac tissue of a child with COVID-19-related multisystem inflammatory syndrome. Lancet Child Adolesc Health.

[43] Faria NR, Mellan TA, Whittaker C, Claro IM, Candido DDS, Mishra S (2021). Genomics and epidemiology of the P.1 SARS-CoV-2 lineage in Manaus, Brazil. Science.

[44] Souza WM, Amorim MR, Sesti-Costa R, Coimbra LD, Brunetti NS, Toledo-Teixeira DA (2021). Neutralisation of SARS-CoV-2 lineage P.1 by antibodies elicited through natural SARS-CoV-2 infection or vaccination with an inactivated SARS-CoV-2 vaccine: an immunological study. Lancet Microbe.

